# Association of Abdominal Incision Length With Gastrointestinal Function Recovery Post-operatively: A Multicenter Registry System-Based Retrospective Cohort Study

**DOI:** 10.3389/fsurg.2021.743069

**Published:** 2021-10-25

**Authors:** Jianning Song, Yingchi Yang, Wenxian Guan, Gang Jin, Yinmo Yang, Lin Chen, Yong Wan, Leping Li, Qingsi He, Wei Zhang, Weiming Zhu, Lei Chen, Dianrong Xiu, Weijun Tian, Daogui Yang, Wenhui Lou, Zhongtao Zhang

**Affiliations:** ^1^Beijing Friendship Hospital Affiliated With Capital Medical University, Beijing, China; ^2^Nanjing Drum Tower Hospital Affiliated With Nanjing University Medical School, Nanjing, China; ^3^Changhai Hospital, Shanghai, China; ^4^Peking University First Hospital, Beijing, China; ^5^The General Hospital of the People's Liberation Army First Medical Center, Beijing, China; ^6^Yantaishan Hospital, Shandong, China; ^7^Shandong Province Hospital, Jinan, China; ^8^Qilu Hospital of Shandong University, Jinan, China; ^9^Jiangxi Province People's Hospital, Nanchang, China; ^10^Nanjing General Hospital of Nanjing Military Command, Nanjing, China; ^11^Qilu Hospital of Shandong University, Qingdao, China; ^12^Peking University Third Hospital, Beijing, China; ^13^General Hospital of Tianjin Medical University, Tianjin, China; ^14^Liaocheng People's Hospital, Liaocheng, China; ^15^Zhongshan Hospital Affiliated With Fudan University, Shanghai, China

**Keywords:** incision length, prolonged post-operative ileus, gastric surgery, colorectal surgery, pancreas-duodenum surgery

## Abstract

**Objective:** To evaluate the influence of the abdominal incision length on the gastrointestinal function recovery post-operatively.

**Background:** Gut motility recovers more quickly after the minimally invasive laparoscopic surgery compared than after the traditional open surgery; however, whether the minimal abdominal incision contributes to the faster gut motility recovery is controversial and lacks solid clinical evidence.

**Methods:** A registry-based secondary cohort analysis was conducted to evaluate the association between the abdominal incision length and gut motility recovery post-operatively based on a multicenter, prospective, and observational study of the prolonged post-operative ileus (PPOI) incidence and the risk factors in the patients with the major abdominal surgery. The incision length, in the centimeters, was the exposure. The primary outcome measures were the PPOI incidence and its association with the incision length. The secondary outcome included the days to the first passage of flatus and the days to the first passage of stool.

**Results:** Overall, 1,840 patients, including 287 (15.7%) patients with the PPOI, were recruited. The PPOI incidence was 17.6% and 13.3% in the long-incision (>18 cm) and short-incision patients ( ≤ 18 cm), respectively. The incidence of the PPOI increased by 1.1% (1.0–1.1) by each centimeter increment of the incision length after adjusting for the confounding factors. In comparison to the short-incision patients, the long-incision patients had prolonged passage of stool (4.46 vs. 4.95 days, *p* < 0.001). Each centimeter increment of the incision length contributed to a 2% increased risk of delay in the first bowel movement [hazard ratio (HR) 0.980 (0.967, 0.994)].

**Conclusion:** A long abdominal incision length independently contributed to the prolonged gut function recovery post-operatively mainly by delaying the time to the first bowel movement, but not influencing the time to first passage of flatus.

## Introduction

Abdominal alimentary tract surgery results in the post-operative ileus (POI), which is defined as the transient cessation of the coordinated bowel motility after surgery. Resolution of POI is important for shortening the post-operative hospitalization in the era of enhanced recovery after surgery (ERAS). As a minimally invasive modality, laparoscopic surgery greatly enhances the recovery of the patient after surgery. Many studies have shown that the laparoscopic surgery is associated with decreased risk of prolonged ileus (1, 2). Patients have a faster bowel motility recovery and an earlier ability to tolerate an oral diet compared with the traditional open surgery. However, the reason for this benefit remains undefined. It is thought that some advantages of the laparoscopic surgery, including less handling of the intestine and the fine dissection of organs, could contribute to the return of the faster bowel function. The short abdominal incision length is a prominent feature of the laparoscopic surgery compared to the open surgery. Some comparative studies by using the animal models have indicated that the magnitude of the abdominal incision affects the duration of POI (3). The minimal incision independently contributes to the faster bowel motility recovery in the patients with major abdominal surgery that remains controversial. The association of the abdominal incision length with POI needs to be explained.

Patient with the prolonged post-operative ileus (PPOI) cohort study was conducted primarily to investigate the incidence of the PPOI in the patients undergoing open gastrointestinal (GI) surgery. The PPOI was defined as POI lasting longer than the regular resolution period, which is generally the post-operative days 3–4. There are definite PPOI diagnostic criteria based on the systematic review and global survey (4). The PPOI is diagnosed if two or more of the following criteria are met on or after day 4 post-operatively: lack of passage of flatus and/or stool, nausea and vomiting, inability to tolerate oral diet, abdominal distension, and diffuse dilated bowel in a CT scan. The strategy for evaluating the bowel function associated with the PPOI is more comprehensive than the passage of flatus and stool. The PPOI has a profound effect on the course of post-operative recovery. It is recognized as a pathological entity that results in an increased length of the hospital stay and healthcare costs (5).

We designed a retrospective cohort study based on the PPOI registry cohort to investigate the effect of the abdominal incision length on the GI function recovery post-operatively. The variable under study was the abdominal incision length. The PPOI incidence was the primary outcome and the time to the first passage of flatus and the time to the first passage of stool were evaluated as the secondary outcomes.

## Materials and Methods

### Study Design

This study was a secondary analysis of the PPOI cohort. This cohort was part of a prospective, multicenter, and observational cohort study of the PPOI incidence and risk factors in the patients with major abdominal surgery. Overall, 2,083 patients from 22 hospitals in the different areas of China were registered in the dataset. The patient series were consecutively admitted to the hospitals between October 26, 2016, and November 5, 2018. All the patients underwent the open major GI surgery including gastric surgery, colorectal surgery, and pancreaticoduodenectomy surgery. They were followed up after surgery until discharged from the hospitals. A consultant surgeon supervised the data collection at each center ensuring that the data were collected in accordance with the protocol. The final dataset was audited by an independent data validator with 97% ascertainment and 91% data accuracy.

The PPOI cohort was approved by the Ethics Committee of Beijing Friendship Hospital (approve code: 2016-P2-064-01). This cohort was registered on the Chinese Clinical Trial Registry website and the registry ID is ChiCTR-IOC-16009955 (http://www.chictr.org.cn/showproj.aspx?proj=16810).

### Exposure Variable

The abdominal incision length was measured in the centimeters by using a sterile ruler after the open abdominal surgery. The patients were divided into a long incision length group (>18 cm) and a short incision length group ( ≤ 18 cm) according to the mean of all the incision lengths (18 cm). When the logistic regression was performed, the exposure variable incision length acted as a continuous variable. The continuous incision length was recorded as a categorical variable according to the quartile number. Dealing with the categorical incision length could provide the evidence whether there was a linear trend of the odds ratio (OR) across all the four quartiles.

### Outcome

The primary outcome was the PPOI incidence after the open abdominal surgery. POI after open abdominal surgery was recognized and it generally returned during the post-operative days 3–4. Delayed recovery of POI has been recognized as a pathological entity and named as the PPOI. The PPOI diagnostic criteria follow the definition proposed by the researchers at the Auckland University, which was determined to be based on the systematic review and global survey (4). The PPOI is diagnosed if two or more of the following criteria are met on or after day 4 post-operatively without prior resolution of POI: (1) nausea/vomiting over 12 h, whereby nausea was measured on a numerical rating scale with 1 indicating least nausea and 10 indicating severe nausea and a rating score >4 indicated positivity; (2) inability to tolerate an oral diet over 24 h or the diet volume was <25% of the normal volume over the two previous meals; (3) absence of flatus or stool over 24 h and if a stoma was performed, absence of gas or stool in the ostomy bags; (4) abdominal distension defined as an increase in the waist circumference and hollow sound through percussion; and (5) radiological confirmation via CT scan or X-ray showing a dilated fluid-filled stomach or bowel.

The secondary outcomes were the days to the first passage of flatus and the days to the first passage of stool. These two variables were counted as the days from surgery date to the date of first flatus and bowel movement and they are the traditional indicators of the bowel motility recovery.

### Covariates

Additional variables that had been previously thought to relate to the bowel motility and the variables that could influence the abdominal incision length were recorded.

The patient variables included the age, sex, body mass index (BMI), primary diagnosis, and the American Society of Anesthesiologists (ASA) grade.

The surgery-related variables included the National Nosocomial Infections Surveillance (NNIS) risk index, specific surgery name, surgical duration (min), blood loss during surgery, blood transfusion during surgery, different centers, year of surgery, and type of abdominal incision. Patients who underwent the Hartmann's procedure, abdominoperineal resection, or wedge resection were recorded as having an anastomosis number of zero. In total, 42 patients did not have anastomosis in our dataset.

### Statistical Analysis

The patient characteristics are presented as the mean for the continuous data and count/percentage for the categorical data. A *t*-test (normal distribution) or Kruskal–Wallis rank-sum test (non-normal distribution) for the continuous data and a chi-squared test or Fisher's exact test for the categorical data were used to compare the basal characteristics distribution between the exposure group and the non-exposure group.

Univariate and multivariate logistic regressions were performed to examine whether the abdominal incision length had an independent effect on the PPOI. The covariates such as surgery-related characteristics and the status variables of the patient were included in the regression model for adjustment. The effect estimates are reported as ORs with the corresponding 95% CIs.

The days to the first passage of flatus and the days to the first bowel movement were analyzed by using the Cox proportional hazards regression model. The effect estimates are reported as the hazard ratios (HRs) with the corresponding 95% CIs.

Subgroup analyses were performed for the different centers and years of surgery to demonstrate the possible bias effect. The five largest sample size centers were applied for the subgroup analysis.

The threshold of two-sided statistical significance was set at *p* < 0.05 a priori. All the analyses were performed with SPSS Statistics version 26.0 and R version 4.1.1(http://www.R-project.org).

## Results

**Figure 1:**
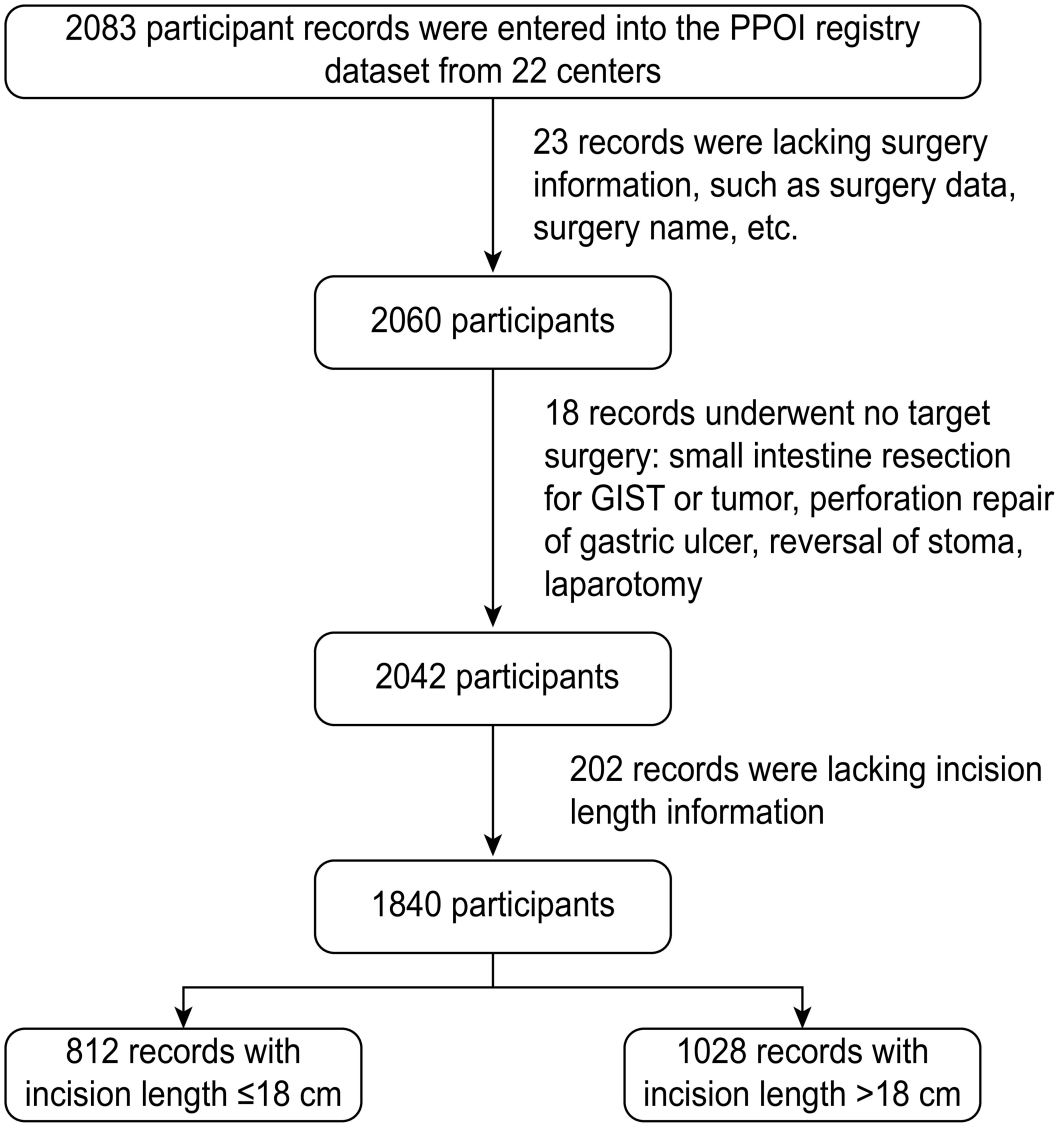
Flowchart of the participants inclusion and exclusion.

The flowchart gives an overview of the inclusion and exclusion criteria of the patient. A total of 2,083 participants from the 22 centers were registered in the PPOI cohort and 243 patients were excluded for the different reasons (Figure 1). Overall, 1,840 patients were included in this secondary cohort analysis. During the hospital stay follow-up period, 287 patients developed the PPOI and the PPOI incidence was 15.7%.

All the basal characteristic variables had more than 90% completed data collection (Table 1). The included patients were divided into the short-incision group ( ≤ 18 cm) and the long-incision group (>18 cm). The patient status-related characteristics, including age, sex, and BMI, were balanced between the long-incision group and the short-incision group (Table 1). However, there were more patients with the ASA grade P3 (22 vs. 14.1%) and the fewer patients with the ASA grade P2 (71 vs. 79.9%) in the long-incision group compared with the short-incision group. The NNIS score had a different distribution between the two groups (*p* < 0.001). A total of 30.6% of the 413 patients with the short-incision group had an NNIS score of zero, while 17.5% of the patients with the long-incision group had an NNIS score of zero.

**Table 1:** Baseline characteristics of the participants (*n* = 1,840).

	**Overall**	**Data completion**	**Incision length ≤18 cm**	**Incision length >18 cm**	***p***
N	1,840		812	1028	
Age, mean ± SD, year	60.85 ± 13.25	99.84%	60.24 ± 14.22	61.33 ± 12.42	0.079
Sex (%)		99.89%			0.734
Female	652 (35.5)		292 (36.0)	360 (35.1)	
Male	1,186 (64.5)		520 (64.0)	666 (64.9)	
BMI, mean ± SD, kg/m^2^	23.17 ± 3.23	89.51%	23.29 ± 3.27	23.09 ± 3.21	0.211
ASA (%)		98.21%			<0.001
1	114 (6.3)		48 (6.0)	66 (6.6)	
2	1,354 (74.9)		642 (79.9)	712 (71.0)	
3	334 (18.5)		113 (14.1)	221 (22.0)	
4	4 (0.2)		1 (0.1)	3 (0.3)	
5	1 (0.1)		0 (0.0)	1 (0.1)	
NNIS (%)		88.97%			<0.001
0	386 (23.6)		232 (30.6)	154 (17.5)	
1	810 (49.5)		340 (44.8)	470 (53.5)	
2	392 (23.9)		156 (20.6)	236 (26.9)	
3	49 (3.0)		31 (4.1)	18 (2.1)	
Surgery (%)		100.00%			<0.001
Gastric	740 (40.2)		303 (37.3)	437 (42.5)	
Pancreas-duodenum	524 (28.5)		156 (19.2)	368 (35.8)	
Colorectal	576 (31.3)		353 (43.5)	223 (21.7)	
Anastomosis number (%)		91.79%			<0.001
0	41 (2.4)		23 (3.1)	18(1.9)	
1	699 (41.4)		393 (53.5)	306 (32.0)	
2	496 (29.4)		181 (24.7)	315 (33.0)	
3	446 (26.4)		134 (18.3)	312 (32.7)	
4	7 (0.4)		3 (0.4)	4 (0.4)	
Transfusion during surgery (%)		95.54%			0.242
No	1,481 (84.2)		669 (85.4)	812 (83.3)	
Yes	277 (15.8)		114 (14.6)	163 (16.7)	
Surgery duration, mean ± SD, min	216.90 ± 91.24	99.29%	198.93 ± 82.26	231.11 ± 95.43	<0.001
PCA (%)		99.78%			<0.001
No	673 (36.7)		216 (26.6)	457 (44.6)	
Yes	1,163 (63.3)		595 (73.4)	568 (55.4)	
Post-operative transfusion (%)		98.21%			0.996
No	1,626 (90.0)		715 (90.1)	911 (89.9)	
Yes	181 (10.0)		79 (9.9)	102 (10.1)	
Time to first flatus, mean ± SD, days	3.47 ± 2.04	92.01%	3.49 ± 2.00	3.45 ± 2.07	0.736
Time to first bowel movement, mean ± SD, days	4.73 ± 2.48	90.27%	4.46 ± 2.58	4.95 ± 2.38	<0.001
PPOI (%)		99.35%			0.013
No	1,541 (84.3)		700 (86.7)	841 (82.4)	
Yes	287 (15.7)		107 (13.3)	180 (17.6)	

*A t-test (normal distribution) or the Kruskal–Wallis rank-sum test (non-normal distribution) was used for the continuous data and a chi-squared test or Fisher's exact test was used for the categorical data*.

*PCA, patient-controlled analgesia*.

Incision length is dependent on the surgical organ and surgical type. Our data showed that the incision length was closely related to the surgical organ, anastomosis number, and surgical duration (Table 1). There were more patients who underwent gastric (42.5 vs. 37.3%) or pancreas–duodenum (35.8 vs. 19.2%) surgery in the long-incision group compared with the short-incision group. A total of 43.5% of the patients with the short-incision group underwent colorectal surgery, while it was 21.7% of the patients with the long-incision group underwent colorectal surgery (*p* < 0.001). The surgical duration in the long-incision group was 231.11 ± 95.43 min, which was longer than the 198.93 ± 82.26 min in the short-incision group (*p* < 0.001). In the long-incision group, 33 and 32.7% of the patients had two and three anastomoses, respectively. The corresponding values were 24.7 and 18.3% in the short-incision group, respectively (*p* < 0.001). In this cohort study, 73.4% of the patients in the short-incision group received patient-controlled analgesia (PCA) compared to 55.4% in the long-incision group (*p* < 0.001).

The primary outcome of the PPOI incidence was 17.6% in the long-incision group and 13.3% in the short-incision group (*p* = 0.013) (Table 1). With respect to the secondary outcome, the overall patients delivered the first flatus on the post-operative days 3.47 ± 2.04 and the first bowel movement on the post-operative days 4.73 ± 2.48. In comparison to the patients with the short incisions, those patients with the long incisions had delayed the first passage of stool (4.46 vs. 4.95 days, *p* < 0.001). However, the time to the first flatus was not significantly different between the long- and short-incision groups (3.49 vs. 3.45 days, *p* = 0.736).

The logistic univariate analysis showed that the OR of incision length was 1.06 (95% CI 1.02–1.09) for the PPOI (Table 2). The univariate analysis also identified the several risk factors for the PPOI including age, organ surgery, transfusion in surgery, and patient-controlled analgesia. Multivariate logistic regression including all these confounding factors was performed to infer an independent relationship between the incision length and the PPOI. The analysis data showed that the incision length was an independent predictor for the PPOI (OR 1.07, 95% CI 1.03–1.11). The possibility increased nearly 10% by each centimeter increment in the incision length independently. Different regression models that adjusted different sets of the confounders confirmed the result regardless of the continuous or categorical incision length (Supplementary Table 1).

**Table 2:** Univariate and multivariate logistics analysis for the prolonged post-operative ileus (PPOI).

		**Univariate**		**Multivariate**	
**Covariates**	**Statistics**	**OR (95%CI)**	***p*-value**	**OR (95%CI)**	***p*-value**
Incision length, mean ±SD, cm	17.2 ± 4.1	1.06 (1.02, 1.09)	0.0008	1.07 (1.03, 1.11)	0.0002
Age, mean ± SD, year	60.8 ± 13.2	1.02 (1.01, 1.03)	0.0053	1.02 (1.00, 1.03)	0.005
Gender (%)					
Female	652 (35.5%)	Reference			
Male	1,186 (64.5%)	1.07 (0.82, 1.40)	0.6155		
BMI, mean ± SD, kg/m^2^	23.2 ± 3.2	1.00 (0.96, 1.04)	0.9121		
NNIS (%)					
0	386 (23.6%)	Reference			
1	810 (49.5%)	1.17 (0.85, 1.65)	0.3435		
2	392 (23.9%)	0.93 (0.62, 1.38)	0.7045		
3	49 (3.0%)	0.37 (0.09, 1.05)	0.1035		
ASA (%)					
1	114 (6.3%)	Reference			
2	1,354 (74.9%)	1.49 (0.85, 2.83)	0.1879		
>3	339 (18.8%)	1.49 (0.80, 2.95)	0.2286		
Surgical organ (%)					
Gastric	740 (40.2%)	Reference		Reference	
Pancreas-duodenum	524 (28.5%)	0.84 (0.62, 1.14)	0.2758	0.80 (0.58, 1.10)	0.170
Colorectal	576 (31.3%)	0.70 (0.51, 0.95)	0.0223	0.68 (0.49, 0.94)	0.023
Surgery duration, mean ± SD, min	216.9 ± 91.2	1.00 (0.99, 1.00)	0.4262		
Transfusion during surgery (%)					
No	1,481 (84.2%)	Reference		Reference	
Yes	277 (15.8%)	1.41 (1.00, 1.95)	0.0425	1.41 (1.00, 1.96)	0.0458
Post-operative transfusion (%)					
No	1,626 (90.0%)	Reference			
Yes	181 (10.0%)	1.09 (0.71, 1.62)	0.6904		
Anastomosis number (%)					
0	41 (2.4%)	Reference			
1	699 (41.4%)	1.08 (0.7, 2.91)	0.867		
2	496 (29.4%)	1.11 (0.48, 3.02)	0.815		
>3	453 (26.8%)	1.14 (0.50, 3.10)	0.773		
PCA (%)					
No	673 (36.7%)	Reference		Reference	
Yes	1,163 (63.3%)	1.28 (0.98, 1.68)	0.0698	1.55 (1.15, 2.09)	0.003
Incision type (%)					
Upper midline	1,233 (71.5%)	Reference		Reference	
Lower midline	264 (15.3%)	0.84 (0.60, 1.15)	0.290	0.57 (0.08, 2.33)	0.490
Right side	142 (8.2%)	0.54 (0.29, 0.93)	0.035	0.36 (0.05, 1.38)	0.197
Left side	85 (4.9%)	0.73 (0.36, 1.35)	0.352	0.53 (0.08, 2.04)	0.421
Year of surgery (%)					
2016	71 (3.9%)	Reference		Reference	
2017	1,439 (78.2%)	1.49 (0.74, 3.41)	0.299	1.69 (0.76, 4.51)	0.237
2018	330 (17.9%)	1.22 (0.57, 2.91)	0.625	1.36 (0.57,3.57)	0.517

We were curious about whether the different incision types might have the different influences on the PPOI. Most of the included patients had a vertical incision: upper midline incision (*n* = 1,233), lower midline incision (*n* = 264), incision per right rectus abdominis (*n* = 142), and per left rectus abdominis (*n* = 85). Sixteen patients with subcostal, transverse, or other incisions were ascribed to the miscellaneous surgery. The univariate logistic regression showed that an incision per right rectus abdominis was less likely to develop the PPOI compared with an upper midline incision (OR 0.54 95% CI 0.29–0.93). However, the multivariate logistic regression demonstrated a non-significant difference among all the four types of the incisions (*p* > 0.05, Table 2).

The multivariate logistic regression displayed the several other independent predictors for the PPOI (Table 2). The OR of age for the PPOI was 1.02 (95% CI 1.00–1.03). Colorectal (OR 0.68 95% CI 0.49–0.94) and pancreas–duodenum (OR 0.80 95% CI 0.58–1.10) surgery had a lower possibility of developing the PPOI compared with the gastric surgery. Transfusion during surgery (OR 1.41 95% CI 1.00–1.96) and usage of PCA (OR 1.55 95% CI 1.15–2.09) increased the chance of developing the PPOI.

The secondary outcome analysis showed that the incision length did not have a significant negative effect on the time to the first passage of flatus (HR 0.99 95% CI 0.98–1.01) (Table 3). However, a long incision length was independently associated with a prolonged time to the first bowel movement (HR 0.98 95% CI 0.97–0.99) and each centimeter increment of the incision length contributed to a 2% increased risk of delaying the first bowel movement (Table 4). A significant relationship existed regardless of the incision type (Table 4). The multivariate Cox HR analysis showed several independent risk factors for the delayed passage of stool. Age (HR 0.99 95% CI 0.98–0.99) and the NNIS score were negatively correlated with the time to the first bowel movement (Table 4).

**Table 3:** Univariate and multivariate Cox analysis for the days to the first flatus.

		**Univariate**		**Multivariate**	
**Covariates**	**Statistics**	**HR (95%CI)**	***p*-value**	**HR (95%CI)**	***p*-value**
Incision length, mean ± SD, cm	17.1 ± 4.0	0.996 (0.984,1.008)	0.477	0.99 (0.98, 1.01)	0.4962
Age, mean ± SD, years	61.7 ± 11.3	0.997 (0.993,1.002)	0.207		
Gender (%)					
Female	601 (35.6%)	Reference			
Male	1,089 (64.4%)	0.967 (0.875,1.068)	0.507		
BMI, mean ± SD, kg/m^2^	23.2 ± 3.2	0.984 (0.968,0.999)	0.038	0.99 (0.97, 1.00)	0.2670
NNIS (%)					
0	361 (23.6%)	Reference		Reference	
1	755 (49.3%)	1.178 (1.039, 1.336)	0.011	1.19 (1.03, 1.38)	0.0178
2	367 (24.0%)	1.028 (0.889, 1.189)	0.706	1.12 (0.93, 1.36)	0.2220
3	48 (3.1%)	1.566 (1.159, 2.117)	0.004	1.81 (1.27, 2.57)	0.0009
ASA (%)					
1	93 (5.5%)	Reference		Reference	
2	1,274 (75.6%)	0.789 (0.639,0.974)	0.027	0.84 (0.66, 1.07)	0.1608
3	318 (18.9%)	0.717 (0.569, 0.903)	0.005	0.71 (0.54, 0.95)	0.0203
Surgical organ (%)					
Gastric	687 (40.7%)	Reference		Reference	
Pancreas-duodenum	475 (28.1%)	1.028 (0.914, 1.156)	0.646	0.91 (0.78, 1.05)	0.2031
Colorectal	528 (31.2%)	1.202 (1.072, 1.346)	0.002	1.12 (0.99, 1.28)	0.0790
Surgery duration, min	213.1 ± 87.5	1.000 (0.999, 1.000)	0.833	1.00 (0.99, 1.00)	0.0582
Transfusion during surgery (%)					
No	1,395 (84.8%)	Reference		Reference	
Yes	250 (15.2%)	0.856 (0.748, 0.980)	0.024	0.93 (0.79, 1.10)	0.3893
Post-operative transfusion (%)					
No	1,508 (90.0%)	Reference		Reference	
Yes	168 (10.0%)	0.831 (0.708, 0.975)	0.023	0.90 (0.74, 1.08)	0.259
Anastomosis number (%)					
0	37 (2.3%)	Reference			
1	650 (40.6%)	0.93 (0.67, 1.30)	0.672		
2	473 (29.5%)	0.94 (0.68, 1.32)	0.736		
>3	442 (27.6%)	0.91 (0.65, 1.27)	0.579		
PCA (%)					
No	576 (34.1%)	Reference		Reference	
Yes	1,113 (65.9%)	0.914 (0.826, 1.011)	0.079	0.91 (0.79, 1.04)	0.1455
Incision type					
Upper midline	1,233	Reference		Reference	
Lower midline	264	1.13 (1.00, 1.28)	0.054	1.18 (0.52, 2.64)	0.691
Right side	142	1.08 (0.90, 1.29)	0.420	1.33 (0.58, 3.02)	0.503
Left side	85	1.39 (1.11, 1.75)	0.004	1.62 (0.76, 3.45)	0.212
Year of surgery					
2016	71	Reference		Reference	
2017	1,439	1.09 (0.85, 1.39)	0.503	1.20 (0.90, 1.61)	0.215
2018	330	1.19 (0.92, 1.55)	0.192	1.54 (1.13, 2.10)	0.007

**Table 4:** Univariate and multivariate Cox analysis for the days to the first bowel movement.

**Variables**	**Statistics**	**HR (95%CI)**	***p*-value**	**HR (95%CI)**	***p*-value**
Incision length, mean ± SD, cm	17.1 ± 4.0	0.983 (0.973,0.994)	0.003	0.98 (0.97, 0.99)	0.005
Age, mean ± SD, years	61.69 ± 11.3	0.993 (0.989,0.998)	0.003	0.99 (0.98, 0.99)	0.0078
Gender (%)					
Female	593 (35.7%)	Reference			
Male	1,066 (64.3%)	0.947 (0.856,1.047)	0.289		
BMI, mean ± SD, kg/m^2^	23.17 ± 3.2	0.994 (0.978,1.009)	0.429		
NNIS (%)					
0	359 (24.1%)	Reference		Reference	
1	737 (49.4%)	0.850 (0.749,0.964)	0.011	0.91 (0.79, 1.04)	0.1576
2	364 (24.4%)	0.698 (0.603,0.808)	<0.0001	0.76 (0.65, 0.89)	0.0008
3	31 (2.1%)	0.504 (0.348,0.728)	0.000	0.45 (0.30, 0.66)	<0.001
ASA (%)					
1	92 (5.6%)	Reference			
2	1,250 (76.1%)	1.174 (0.950,1.451)	0.139		
3	301 (18.3%)	0.938 (0.743,1.185)	0.593		
Surgical organ (%)					
Gastric	670 (40.4%)	Reference		Reference	
Pancreas-duodenum	479 (28.9%)	1.078 (0.958,1.212)	0.211	1.13 (0.99, 1.30)	0.0772
Colorectal	510 (30.7%)	1.124 (1.001,1.261)	0.048	1.10 (0.97, 1.24)	0.1416
Surgery duration, mean ± SD, min	214.0 ± 89.1	0.999 (0.998,0.999)	<0.0001	1.00 (0.99, 1.00)	0.438
Transfusion during surgery (%)					
No	1,365 (84.7%)	Reference			
Yes	247 (15.3%)	0.885 (0.773,1.014)	0.781		
Post-operative transfusion (%)					
No	1,477 (89.8%)	Reference			
Yes	168 (10.2%)	0.936 (0.798,1.099)	0.420		
Anastomosis number (%)					
0	34 (2.2%)	Reference			
1	634 (40.6%)	0.98 (0.69,1.38)	0.886		
2	453 (29.1%)	0.93 (0.66,1.32)	0.676		
>3	439 (28.1%)	1.01 (0.71,1.44)	0.939		
PCA (%)					
No	573 (34.6%)	Reference		Reference	
Yes	1,085 (65.4%)	1.152 (1.041,1.275)	0.002	1.10 (0.98, 1.24)	0.1122
Incision type					
Upper midline	1,233	Reference		Reference	
Lower midline	264	1.05 (0.93, 1.19)	0.463	0.82 (0.44, 1.52)	0.520
Right side	142	1.20 (1.00, 1.44)	0.043	1.00 (0.54, 1.83)	0.989
Left side	85	0.93 (0.73, 1,19)	0.549	0.76 (0.42, 1.36)	0.352
Year of surgery					
2016	71	Reference		Reference	
2017	1,439	1.18 (0.92, 1.52)	0.187	1.16 (0.89, 1.54)	0.288
2018	330	1.16 (0.88, 1.51)	0.291	1.19 (0.89, 1.60)	0.243

**Figure 2:**
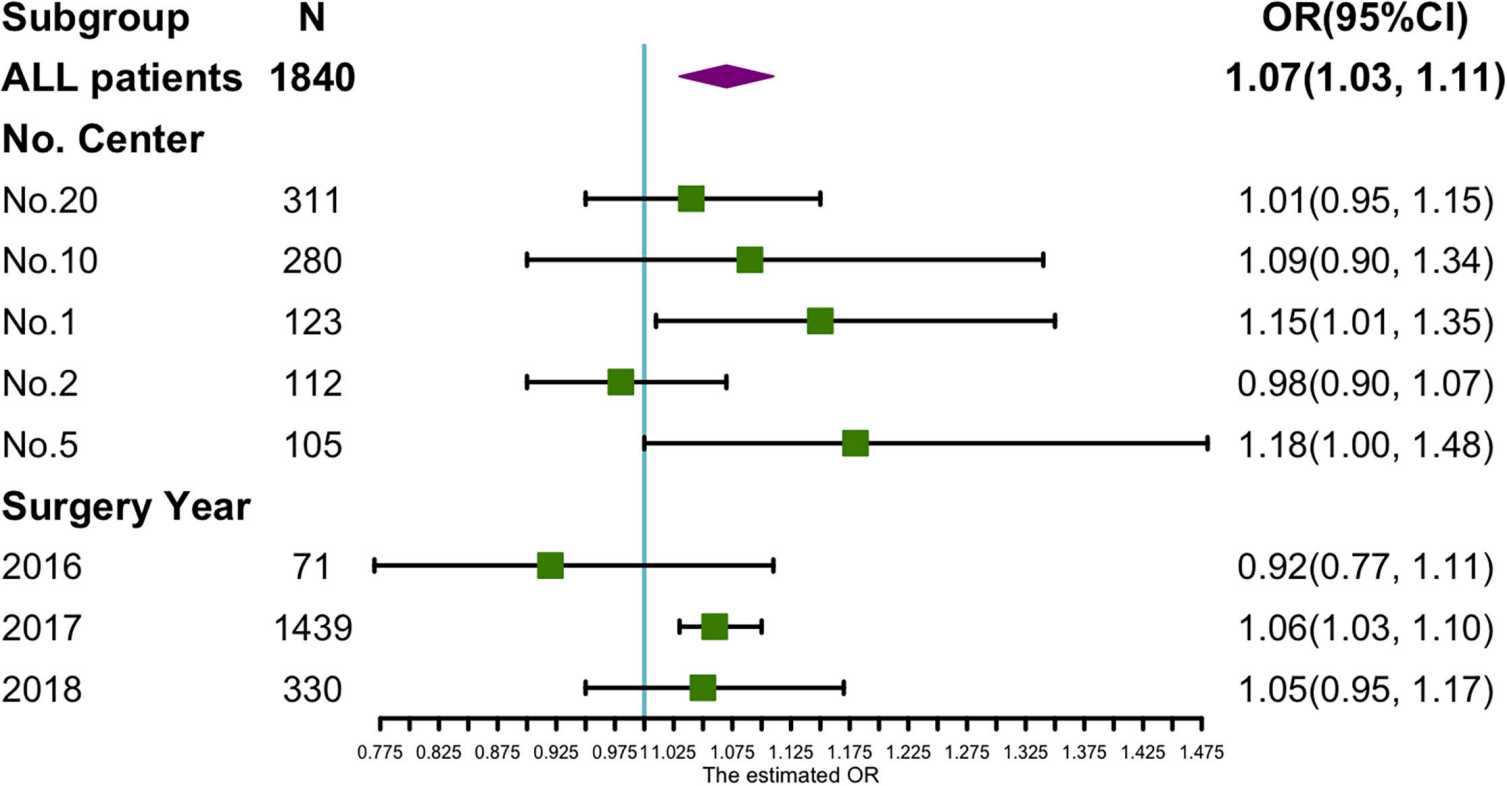
Sensitivity analysis of incision length on PPOI incidence in subgroups of different surgery years and hospital centers.

Sensitivity analyses were performed for the different centers and years of surgery. Five out of 22 centers recruited more than 100 participants. Logistic regression for the correlation of the PPOI and incision length was applied in the five largest centers. Figure 2 shows that there was a positive correlation between incision length and PPOI incidence in all five subcenters, but three of them did not reach significance. With respect to the year of surgery of the subgroups, the data showed that the incision length was independently positively related to the PPOI in the 2,017 subgroups (OR 1.06 95% CI 1.03–1.10), which included the largest number of the participants (*n* = 1,439). However, the OR did not reach significance in the smallest sample size of 2,016 subgroups (Figure 2).

## Discussion

This registry system-based, multicenter, and retrospective cohort study revealed that the incision length was independently positively correlated with the PPOI incidence in the patients who underwent the open abdominal surgery. The incision length independently affected the time to the first bowel movement but not the time to the first flatus. Delayed passage of stool post-operatively contributes to the development of the PPOI.

Our data showed that the PPOI incidence associated with the open abdominal surgery was 15.7%. The subgroup analysis of the surgical organ showed a 13.2% PPOI incidence in the colorectal surgery, 15.5% in the pancreas–duodenum surgery, and 17.8% in the gastric surgery. Recently, the GRACE Collaborative Group For Ileus Study reported the proximate PPOI incidence (15.4%) in the colorectal surgery with the same PPOI diagnostic criteria (6).

The etiology of POI is not well-defined and is thought to be multifactorial: surgical stress response, disruption of intestinal continuity, opioid analgesic use, intestinal manipulation, etc. The inhibitory effect of the opioid analgesics on the GI motility has been well-defined (7). Alvimopan, a novel peripherally-acting μ opioid antagonist without inhibition of the central nervous system, can accelerate the GI motility (5). In our dataset, the patient-controlled analgesia comprising mainly the opioid analgesics was an independent risk factor for the PPOI. It was adjusted in the multivariate model for the incision length effect on the GI motility. Intestinal anastomoses disrupted the integrity of the alimentary tract including the physical and electrical continuity. In a murine model of the small bowel resection, the acute disruptions to the interstitial cells of Cajal (ICC) networks, slow waves, and phasic contractions were found (8). Intestinal manipulation is also an important risk factor for POI. Pancreas–duodenum surgery generally has three anastomoses and is thought to receive a greater degree of the intestinal manipulation. Gastric and colorectal surgeries are thought to have fewer anastomoses and the intestinal manipulations. Our data show that gastric surgery had a higher PPOI incidence (17.8%) followed by the pancreas–duodenum surgery (15.4%) and colorectal surgery (13.2%). This result suggests that the disruption of the intestinal continuity might not play an important role in the bowel function recovery.

There are limited studies regarding the effect of the incision length on the gut motility and most of the results come from the animal models. Well-controlled prospective studies in a mouse model show that the longer and deeper abdominal incisions cause more profound inhibition of GI transit and prolong the period of POI (3). A small sample size study with 40 patients who underwent colectomy showed that the transverse incision length correlated with the time to the first bowel sounds and bowel movement but not the time to the first flatus (9).

Our study focused on the association between the incision length and GI motility recovery. We had a relatively large sample size (*n* = 1,840) based on a multicenter registry system cohort. The included patients underwent the diverse kinds of the major abdominal surgery including gastric surgery, pancreas–duodenum surgery, and colorectal surgery. The large sample size and diversity of the included patients make the conclusion even more universal. The registry system recorded all the variables that might confound the association between the incision length and bowel function recovery such as anastomosis number, surgical duration, surgical organ, and use of opioid analgesic. All these variables were adjusted in the multimodal regression to give an independent relationship between the incision length and bowel function recovery.

Prolonged post-operative ileus, which is a different pathological entity from POI, was our primary outcome. Our data showed that the abdominal incision length was an independent risk factor for the PPOI. The possibility of developing the PPOI increased 10% by each centimeter increment of the incision length after adjusting for all the kinds of the confounders. With respect to the secondary outcome, our data showed that the incision length was independently related to the days to the first bowel movement. Each centimeter increment of the incision length contributed to a 2% increased risk of delay in the first bowel movement. However, the abdominal incision length did not statistically correlate with the time to the first flatus. We also investigated whether the incision type could have a different influence on the gut motility. Four types of the vertical incisions such as upper midline incision, lower midline incision, incision per right rectus abdominis, and per left rectus abdominis constituted 94% of the participants. The results indicated that the gut motility recovery was not significantly different among these four types of the incisions. The negative effect of the incision length on the gut motility recovery existed regardless of the incision type. The bias due to the different hospitals and surgery years was also taken into consideration. Subgroup analyses indicated a bit of heterogeneity across the different hospital centers. However, it might be that the small sample size in 2016 resulted in an unreached significant OR of the incision length for the PPOI.

How the abdominal incision trauma negatively affects the bowel function recovery is not well-understood. It has been suggested that an abdominal incision can activate the adrenergic pathway, which inhibits GI transit (3) through a neurogenic pathway at the early post-operative phase. Adrenergic blockade can improve GI transit after laparotomy in the rats. Neurogenic inhibition of the gut motility diminishes more quickly and the global inflammation inhibition results in the gut dysmotility (10). The peritoneal inflammatory response to an abdominal incision is activated through the neuro-immuno-humoral axis and the inflammatory response retards surgical recovery including prolonged gut motility (11, 12). More specific molecular mechanisms are needed to clarify the detailed relationship between the incision trauma and gut function recovery.

Some limitations of this study should be considered when interpreting the conclusion. This retrospective study was based on a multicenter registry cohort. The missing records might result in the selection bias. A total of 202 patients were excluded because of a lack of the incision length data. We conducted a comparison between the 202 excluded and 1,840 included patients. They were balanced between the age, sex, and BMI. However, there were statistically significant differences among the surgery-related characteristics. This might be the source of the selection bias. In addition, <10% of the data were missing with respect to the secondary outcome of the days to the first flatus and the days to the first bowel movement. Second, since every effort was made to collect the data on the related variables, some confounding covariate data might have been missed. The intestinal inflammatory response following the open abdominal surgery corresponds directly to the gut motility (13). Fluid overload during the perioperative period contributes to the impaired gut motility through edema in the intestinal wall. Electrolyte disturbance involving sodium and potassium also leads to weakened the gut motility (14). All these missed confounding covariates may have influenced the data interpretation. Third, our hospital center subgroup analysis indicated some heterogeneity across the different centers. When considering three out of the five largest sample size centers, it could not be concluded that the incision length was an independent predictor of the PPOI.

## Conclusion

The abdominal incision length was an independent risk factor for the PPOI. The possibility of developing the PPOI increases 10% by each centimeter increment of the incision length after adjusting for all the kinds of confounders. The incision length was independently related to the days to the first bowel movement but not to the days to the first flatus.

## Data Availability Statement

The original contributions presented in the study are included in the article/Supplementary Material, further inquiries can be directed to the corresponding author/s.

## Ethics Statement

The studies involving human participants were reviewed and approved by Ethics Committee of Beijing Friendship Hospital. The patients/participants provided their written informed consent to participate in this study.

## Author Contributions

JS, YingY, WG, GJ, and YinmY: drafted the article and revise it critically for important intellectual content. ZZ made substantial contributions to conception and design and final approval of the article. YingY, WG, GJ, YinmY, LiC, YW, LL, QH, WeiZ, WeimZ, LeC, DX, WT, DY, and WL: made substantial contributions to conception and design and acquisition of data.

## Conflict of Interest

The authors declare that the research was conducted in the absence of any commercial or financial relationships that could be construed as a potential conflict of interest.

## Publisher's Note

All claims expressed in this article are solely those of the authors and do not necessarily represent those of their affiliated organizations, or those of the publisher, the editors and the reviewers. Any product that may be evaluated in this article, or claim that may be made by its manufacturer, is not guaranteed or endorsed by the publisher.
