# Supplementary material for: Association of Abdominal Incision Length With Gastrointestinal Function Recovery Post-operatively: A Multicenter Registry System-Based Retrospective Cohort Study
Source: Front Surg. 2021 Oct 25;8:743069. doi: 10.3389/fsurg.2021.743069 (PMC8575117; doi:10.3389/fsurg.2021.743069)
Supplement: Supplementary file 1 [file Table_1.docx]

**Supplementary Table 1.** Relationship between incision length and PPOI in different models

| Variable | Nonadjusted | | Adjust I | | Adjust II | |
| --- | --- | --- | --- | --- | --- | --- |
|  | OR (95% CI) | *p* | OR (95% CI) | *p* | OR (95% CI) | *p* |
| Incision length, cm | 1.1 (1.0, 1.1) | 0.0008 | 1.1 (1.0, 1.1) | 0.0006 | 1.1 (1.0, 1.1) | 0.0004 |
| Incision length quantile |  |  |  |  |  |  |
| Q1 (≤10 cm) | Reference |  | Reference |  | Reference |  |
| Q2 (>10 cm, ≤15 cm) | 1.63 (0.92, 3.09) | 0.11 | 1.78 (0.92, 3.76) | 0.106 | 2.02 (0.96, 4.67) | 0.078 |
| Q3 (>15 cm, ≤20 cm) | 2.20 (1.29, 4.07) | 0.007 | 2.65 (1.39, 5.51) | 0.005 | 2.99 (1.46, 6.82) | 0.005 |
| Q4 (>20 cm, ≤35 cm) | 3.05 (1.53,6.33) | 0.002 | 3.77 (1.70, 8.81) | 0.001 | 4.14 (1.73, 10.60) | 0.002 |

Adjust I: We adjusted for surgery-related variables, including surgical organ, surgical period, anastomosis number, NNIS, surgery transfusion, postoperative transfusion, and patient-controlled analgesia.

Adjust II: We adjusted for both surgical and patient status-related variables, including age, sex, BMI, ASA, surgical organ, surgical period, anastomosis number, NNIS, surgery transfusion, postoperative transfusion, and patient-controlled analgesia.
